# Immunogenicity and safety of a combined DTPa-IPV/Hib vaccine administered as a three-dose primary vaccination course in healthy Korean infants: phase III, randomized study

**DOI:** 10.1080/21645515.2018.1536588

**Published:** 2018-11-15

**Authors:** Ki Hwan Kim, Chun Soo Kim, Hwang Min Kim, Jong-Duck Kim, Sang Hyuk Ma, Dong Ho Kim, Pyoung-Han Hwang, Ji-Whan Han, Taek-Jin Lee, Joon Hyung Kim, Naveen Karkada, Narcisa Mesaros, Woo-Yun Sohn, Jong-Hyun Kim

**Affiliations:** a Incheon St. Mary’s Hospital, College of Medicine, The Catholic University of Korea Incheon, The Republic of Korea; b Keimyung University School of Medicine, Daegu, The Republic of Korea; c Yonsei University Wonju College of Medicine, Wonju, The Republic of Korea; d Wonkwang University Hospital, Iksan, The Republic of Korea; e Changwon Fatima Hospital, Changwon, The Republic of Korea; f Korea Cancer Center Hospital, Seoul, The Republic of Korea; g Chonbuk National University Hospital, Chonbuk National University Medical School, Jeonju, The Republic of Korea; h Uijeongbu St. Mary’s Hospital, The Catholic University of Korea, Uijeongbu, The Republic of Korea; i Bundang CHA Hospital, Seongnam, The Republic of Korea; j GSK, Seoul, The Republic of Korea; k GSK, Wavre, Belgium; l GSK, Seoul, The Republic of Korea; m St. Vincent’s Hospital, College of Medicine, The Catholic University of Korea, Suwon, The Republic of Korea

**Keywords:** diphtheria, tetanus, acellular pertussis, poliovirus, *Haemophilus influenzae* type b, DTPa-IPV/Hib, immunogenicity, reactogenicity, safety, infants

## Abstract

We assessed the immunogenicity and safety of a three-dose primary vaccination schedule with the combined diphtheria-tetanus-acellular pertussis-inactivated poliovirus/Haemophilus influenzae type b vaccine (DTPa-IPV/Hib) in Korean infants.

In this phase III open-label, multicenter study (NCT01309646), healthy infants aged 42–69 days (randomized 1:1) received three doses of either pentavalent DTPa-IPV/Hib (DTPa-IPV/Hib group) or DTPa-IPV and Hib vaccines administered separately (DTPa-IPV+Hib group) at 2, 4, 6 months of age. The primary objective was to demonstrate non-inferiority of DTPa-IPV/Hib compared to DTPa-IPV+Hib vaccines in terms of immune responses to all vaccine antigens, 1 month post-dose 3. Solicited symptoms (local and general) were recorded during 4 days, and unsolicited adverse events (AEs) during 31 days, after each vaccination. Serious AEs (SAEs) were recorded throughout the study duration.

The immunogenicity of the pentavalent DTPa-IPV/Hib vaccine was non-inferior compared to concomitant administration of DTPa-IPV+Hib vaccines. One month post-dose 3, nearly all infants had antibody levels above the seroprotective thresholds for anti-diphtheria toxoid, anti-tetanus toxoid, anti-polyribosyl-ribitol phosphate, and anti-poliovirus type 1, 2 and 3, and had antibody levels above the seropositive thresholds for anti-pertussis toxoid (PT), anti-filamentous hemagglutinin (FHA) and anti-pertactin (PRN) antibodies. A vaccine response for PT, FHA and PRN was observed in at least 96.7% of study participants. Anti-PRP geometric mean concentrations appeared lower for the DTPa-IPV/Hib group (8.456 µg/mL) than for the DTPa-IPV+Hib group (18.700 µg/mL). In both groups, the most common solicited symptoms were injection site redness and irritability. Fifty-seven SAEs were reported throughout the study; none were considered to be vaccination related.

## Introduction

Routine vaccination against diphtheria, tetanus, pertussis, invasive *Haemophilus influenzae* type b (Hib), and poliomyelitis significantly decreased the incidence of these diseases in many regions of the world. However, the burden remains high in developing countries, where implementation of complex vaccination schedules is challenging due to lack of financial resources and organized vaccination programs.^1^ Using combination vaccines to replace complex schedules have a number of potential benefits, including simplified administration, increased patient and health care acceptance, higher vaccination coverage, and reduction in vaccination costs and number of visits.^2^

A combined pentavalent vaccine against diphtheria, tetanus, acellular pertussis, poliomyelitis, and Hib (DTPa-IPV/Hib; *Infanrix-IPV/Hib*, GSK) was first licensed in 1997.^3^ The vaccine administered as a primary and/or booster vaccination has been shown to be well tolerated and immunogenic in infants in previous studies.^4–9^ Since population-based immune response differences might be observed,^10–14^ further studies may be needed to include multivalent vaccines in the National Immunization Programs of different countries. In Korea, three-dose primary vaccination of infants against diphtheria, tetanus, pertussis, poliomyelitis and Hib is recommended by the Korean Pediatric Society at 2, 4 and 6 months of age.^15^

The present study was performed to evaluate the safety and immunogenicity of the combined DTPa-IPV/Hib vaccine in the Korean population and will provide important insight to healthcare providers and vaccination recommending bodies in the country into the vaccine’s profile in this specific population. We aimed to demonstrate the non-inferiority of the combined DTPa-IPV/Hib vaccine given as a single dose in a three-dose primary vaccination course at 2, 4 and 6 months of age compared with the present standard of care: concomitant administration of DTPa-IPV (*Infanrix-*IPV, GSK) and Hib (*Hiberix*, GSK) vaccines at different injection sites.

## Results

### Study participants

**Figure 1.**
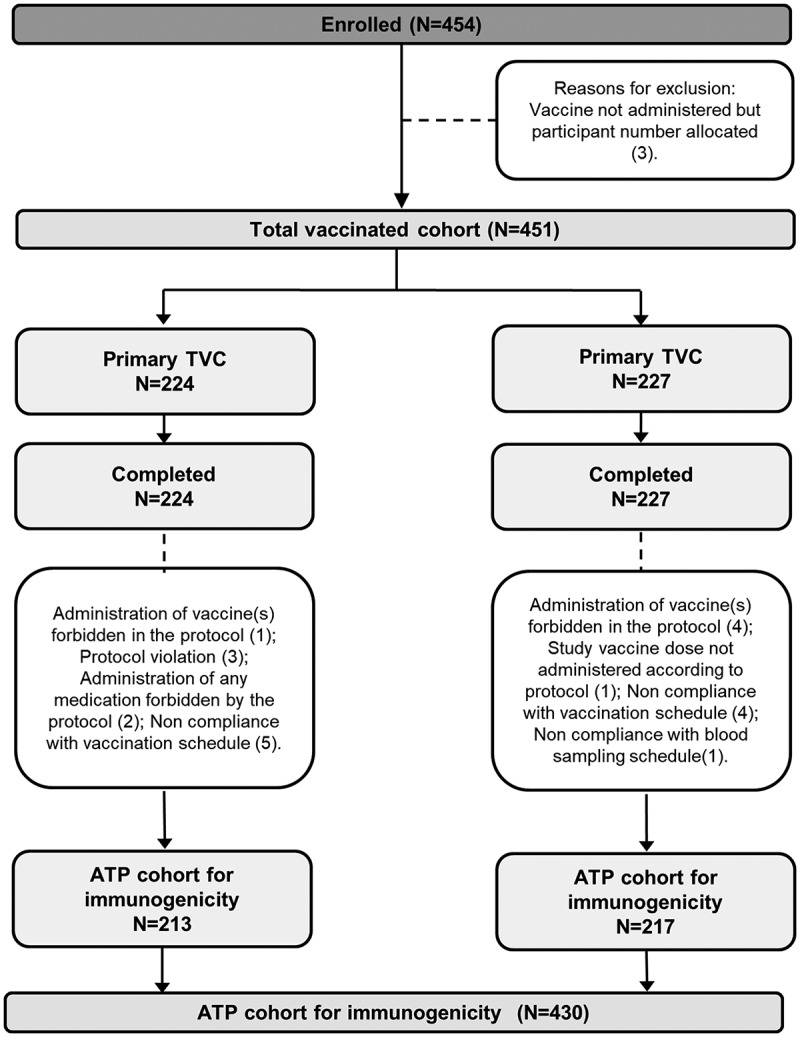
Participant flow diagram. N, number of participants in each group; TVC, total vaccinated cohort; ATP, according-to-protocol. Infants in DTPa-IPV/Hib group received 3 doses of combined diphtheria-tetanus-acellular pertussis-inactivated poliomyelitis and Haemophilus influenzae type b vaccine at 2, 4, 6 months of age and infants in DTPa-IPV+ Hib group received 3 concomitant doses of diphtheria-tetanus-acellular pertussis-inactivated poliomyelitis vaccine and Haemophilus influenzae type b vaccine at 2, 4, 6 months of age.

A total of 454 infants were enrolled in the study. Of these, 224 infants received the combined DTPa-IPV/Hib vaccine (DTPa-IPV/Hib group) and 227 infants received concomitant administration of DTPa-IPV and Hib vaccines (DTPa-IPV+Hib group). All infants were vaccinated according to the Korean national recommended vaccination schedule at 2, 4 and 6 months of age. Four-hundred and fifty-one infants were included in the total vaccinated cohort (TVC) of which 430 infants (213 in the DTPa-IPV/Hib group and 217 in the DTPa-IPV+Hib group) were included in the according-to-protocol (ATP) cohort for immunogenicity (Figure 1). The demographic characteristics were balanced between groups (Table 1).

**Table 1.** Demographic characteristics (total vaccinated cohort).

	DTPa-IPV/Hib group N = 224	DTPa-IPV+ Hib group N = 227	Total N = 451
**Age at dose 1 (weeks**), mean (SD)	8.8 (1.1)	8.8 (1.1)	8.8 (1.1)
**Female/male**, %	43.3/56.7	50.7/49.3	47.0/53.0
Race*, n/%			
Asian – East Asian	224 (100)	226 (99.6)	450 (99.8)

N, number of participants; n/%, number/percentage of participants in a given category; SD, standard deviation.

Infants in DTPa-IPV/Hib group received 3 doses of combined diphtheria-tetanus-acellular pertussis-inactivated poliomyelitis and *Haemophilus influenzae* type b vaccine at 2, 4, 6 months of age and infants in DTPa-IPV+ Hib group received 3 concomitant doses of diphtheria-tetanus-acellular pertussis-inactivated poliomyelitis vaccine and *Haemophilus influenzae* type b vaccine at 2, 4, 6 months of age.

*One participant from the DTPa-IPV+ Hib group had another heritage (not further specified).

### Immunogenicity

The non-inferiority of the DTPa-IPV/Hib vaccine over the separately administered DTPa-IPV and Hib vaccines was demonstrated. The non-inferiority criteria was met when the upper limits (ULs) of the standardized asymptotic 95% confidence intervals (CIs) for the difference between groups (DTPa-IPV+Hib minus DTPa-IPV/Hib group) in terms of percentage of infants seroprotected against diphtheria, tetanus, polyribosyl-ribitol phosphate (PRP) and poliovirus types 1, 2 and 3 were below the pre-defined non-inferiority limit of 10%, and the ULs of the 95% CIs for the group ratios (DTPa-IPV+Hib over DTPa-IPV/Hib group) of anti-pertussis toxoid (PT), anti-filamentous hemagglutinin (FHA) and anti-pertactin (PRN) antibody geometric mean concentrations (GMCs) were below the pre-defined non-inferiority limit of 1.5 (Table 2).

**Table 2.** Group differences in seroprotection/seropositivity rates and adjusted GMC ratio 1M post-dose 3 (ATP immunogenicity cohort).

	DTPa-IPV+ Hib group N = 217	DTPa-IPV/Hib group N = 213	Difference between groups (DTPa-IPV+ Hib minus DTPa-IPV/Hib)
Antibody	n (%)	n (%)	% (95% CI)
Anti-D	217 (100)	213 (100)	0.00 (−1.74–**1.78**)
Anti-T	217 (100)	213 (100)	0.00 (−1.74–**1.78**)
Anti-Polio 1	216 (100)	212 (100)	0.00 (−1.75–**1.78**)
Anti-Polio 2	211 (100)	204 (100)	0.00 (−1.79–**1.85**)
Anti-Polio 3	197 (99.0)	197 (99.5)	−0.50 (−3.14–**1.89**)
Anti-PRP	217 (100)	213 (100)	0.00 (−1.74–**1.78**)
	Adjusted GMC (EL.U/mL)		Adjusted GMC ratio (DTPa-IPV+ Hib over DTPa-IPV/Hib)
			Value (95% CI)
Anti-PT	56.2	54.3	1.04 (0.93–**1.15**)
Anti-FHA	134.6	124.2	1.08 (0.97–**1.21**)
Anti-PRN	134.4	124.9	1.08 (0.96–**1.21**)

M, month; ATP, according-to-protocol; N, maximum number of participants with available results; n (%), number (percentage) of participants with antibody concentrations above the specified cut-off; CI, confidence interval; EL.U/mL, ELISA units per milliliter; anti-D, anti-diphtheria toxoid; anti-T, anti-tetanus toxoid; PRP, polyribosyl-ribitol phosphate; PT, pertussis toxoid; FHA, filamentous hemagglutinin; PRN, pertactin; Adjusted GMC, geometric mean antibody concentration adjusted for baseline concentration.

Infants in DTPa-IPV/Hib group received 3 doses of combined diphtheria-tetanus-acellular pertussis-inactivated poliomyelitis and *Haemophilus influenzae* type b vaccine at 2, 4, 6 months of age and infants in DTPa-IPV+ Hib group received 3 concomitant doses of diphtheria-tetanus-acellular pertussis-inactivated poliomyelitis vaccine and *Haemophilus influenzae* type b vaccine at 2, 4, 6 months of age.

Bolded values indicate that the non-inferiority criteria (upper limits (ULs) of 95% confidence intervals (CIs) of group differences in seroprotection rates [DTPa-IPV+ Hib group minus DTPa-IPV/Hib group] for all antigens ≤ 10% and ULs of 95% CIs of GMC ratios [DTPa-IPV+ Hib group over DTPa-IPV/Hib group] for pertussis antigens ≤ 1.5) have been met.

One month after the third primary vaccination dose, all infants in both groups had antibody levels above the seroprotective thresholds of anti-diphtheria (anti-D), anti-tetanus (anti-T), anti-PRP, and anti-poliovirus types 1 and 2 antibodies, and antibody levels above the seropositive thresholds of anti-PT, anti-FHA, anti-PRN antibodies. At least 99.0% of infants had antibody levels above the seroprotective threshold for anti-poliovirus type 3 antibodies (Table 3).

**Table 3.** Seroprotection/seropositivity rates and GMCs/GMTs at pre-vaccination and 1M post-dose 3 (ATP immunogenicity cohort).

			DTPa-IPV/Hib group						DTPa-IPV+ Hib group					
			Seroprotection/seropositivity						Seroprotection/seropositivity					
Antibody		Time-point	N	n	%	95% CI	GMC/GMT	95% CI	N	n	%	95% CI	GMC/GMT	95% CI
Anti-D ≥ 0.1 IU/mL		Pre	213	29	13.6	9.3–19.0	0.058	0.055–0.061	217	30	13.8	9.5–19.1	0.060	0.056–0.064
		Post	213	213	100	98.3–100	8.096	7.520–8.717	217	217	100	98.3–100	8.692	8.125–9.298
Anti-T ≥ 0.1 IU/mL		Pre	213	64	30.0	24.0–36.7	0.081	0.072–0.090	217	76	35.0	28.7–41.8	0.091	0.080–0.103
		Post	213	213	100	98.3–100	10.259	9.654–10.902	217	217	100	98.3–100	12.421	11.599–13.301
Anti-Polio (≥ 8 ED_50_)	Type 1	Pre	211	104	49.3	42.4–56.2	9.5	8.3–11.0	216	89	41.2	34.6–48.1	9.4	8.0–11.0
		Post	212	212	100	98.3–100	328.8	289.8–373.1	216	216	100	98.3–100	372.7	323.8–428.9
	Type 2	Pre	211	119	56.4	49.4–63.2	10.8	9.4–12.5	214	108	50.5	43.6–57.4	8.8	7.7–9.9
		Post	204	204	100	98.2–100	340.6	295.4–392.6	211	211	100	98.3–100	400.2	347.6–460.7
	Type 3	Pre	211	41	19.4	14.3–25.4	5.5	5.0–6.1	216	31	14.4	10.0–19.7	4.9	4.5–5.3
		Post	198	197	99.5	97.2–100	377.7	322.3–442.6	199	197	99.0	96.4–99.9	465.3	390.3–554.7
Anti-PT (≥ 5 EL.U/mL)	PT	Pre	212	27	12.7	8.6–18.0	3.0	2.8–3.2	216	34	15.7	11.2–21.3	3.1	2.9–3.3
		Post	213	213	100	98.3–100	54.2	50.4–58.3	217	217	100	98.3–100	56.0	51.8–60.5
	FHA	Pre	211	170	80.6	74.6–85.7	10.5	9.3–12.0	214	178	83.2	77.5–87.9	11.7	10.3–13.3
		Post	213	213	100	98.3–100	125.0	115.4–135.4	217	217	100	98.3–100	134.2	124.2–145.0
	PRN	Pre	213	25	11.7	7.7–16.8	2.9	2.7–3.0	217	30	13.8	9.5–19.1	3.1	2.8–3.3
		Post	213	213	100	98.3–100	125.8	116.0–136.5	217	217	100	98.3–100	133.4	123.0–144.6
Anti-PRP	≥ 0.15 µg/mL	Pre	213	91	42.7	36.0–49.7	0.165	0.143–0.191	217	109	50.2	43.4–57.1	0.219	0.184–0.260
		Post	213	213	100	98.3–100	8.456	7.283–9.819	217	217	100	98.3–100	18.700	16.353–21.384
	≥ 1.0 µg/mL	Pre	213	14	6.6	3.6–10.8	0.165	0.143–0.191	217	30	13.8	9.5–19.1	0.219	0.184–0.260
		Post	213	208	97.7	94.6–99.2	8.456	7.283–9.819	217	217	100	98.3–100	18.700	16.353–21.384

M, month; ATP, according-to-protocol; N, number of participants with available results; n (%), number (percentage) of participants with concentration ≥ required threshold; GMC/GMT, geometric mean antibody concentration/titer; CI, confidence interval; EL.U/mL, ELISA units per milliliter; ED_50_, median effective dose; IU/mL, international units per milliliter; Pre, blood sample collected before the first dose of the primary vaccination course; Post, blood sample collected after the third dose of the primary vaccination course; anti-D, anti-diphtheria; anti-T, anti-tetanus; PT, pertussis toxoid; FHA, filamentous hemagglutinin; PRN, pertactin; PRP, polyribosyl-ribitol phosphate.

The DTPa-IPV/Hib group received the combined DTPa-IPV/Hib vaccine at 2, 4 and 6 months of age.

The DTPa-IPV+ Hib group received the DTPa-IPV and Hib vaccines separately at 2, 4 and 6 months of age.

**Table 4.** Vaccine response rate to anti-PT, anti-FHA and anti-PRN antibodies 1M post-dose 3 (ATP immunogenicity cohort).

	% Vaccine response (95% CI)	
Antibody	DTPa-IPV/Hib group (CI 95%)	DTPa-IPV+ Hib group (CI 95%)
Anti-PT	99.5 (97.4–100)	99.5 (97.4–100)
Anti-FHA	98.1 (95.2–99.5)	96.7 (93.4–98.7)
Anti-PRN	100 (98.3–100)	99.5 (97.5–100)

M, month; ATP, according-to-protocol; %, percentage of infants with vaccine response; CI, confidence interval; PT, pertussis toxoid; FHA, filamentous hemagglutinin; PRN, pertactin.

Infants in DTPa-IPV/Hib group received 3 doses of combined diphtheria-tetanus-acellular pertussis-inactivated poliomyelitis and *Haemophilus influenzae* type b vaccine at 2, 4, 6 months of age and infants in DTPa-IPV+ Hib group received 3 concomitant doses of diphtheria-tetanus-acellular pertussis-inactivated poliomyelitis vaccine and *Haemophilus influenzae* type b vaccine at 2, 4, 6 months of age.

Vaccine response was defined as an antibody concentration ≥ 5 EL.U/mL post-dose 3 for initially seronegative infants, or ≥ 1-fold increase the pre-vaccination antibody concentration for initially seropositive infants.

Although anti-D, anti-poliovirus types 1, 2 and 3, and anti-PT antibody GMCs/geometric mean titers (GMTs) were within clinically acceptable ranges in both groups, anti-T and anti-PRP GMCs appeared numerically lower in the DTPa-IPV/Hib group compared to the DTPa-IPV+Hib group, based on non-overlapping 95% CIs (10.259 international units (IU)/mL versus 12.421 IU/mL; and 8.456 µg/mL versus 18.700 µg/mL) (Table 3).

The vaccine response to pertussis antigens was comparable between the groups, with at least 96.7% of infants showing vaccine responses to PT, FHA and PRN (Table 4).

### Safety

**Figure 2.**
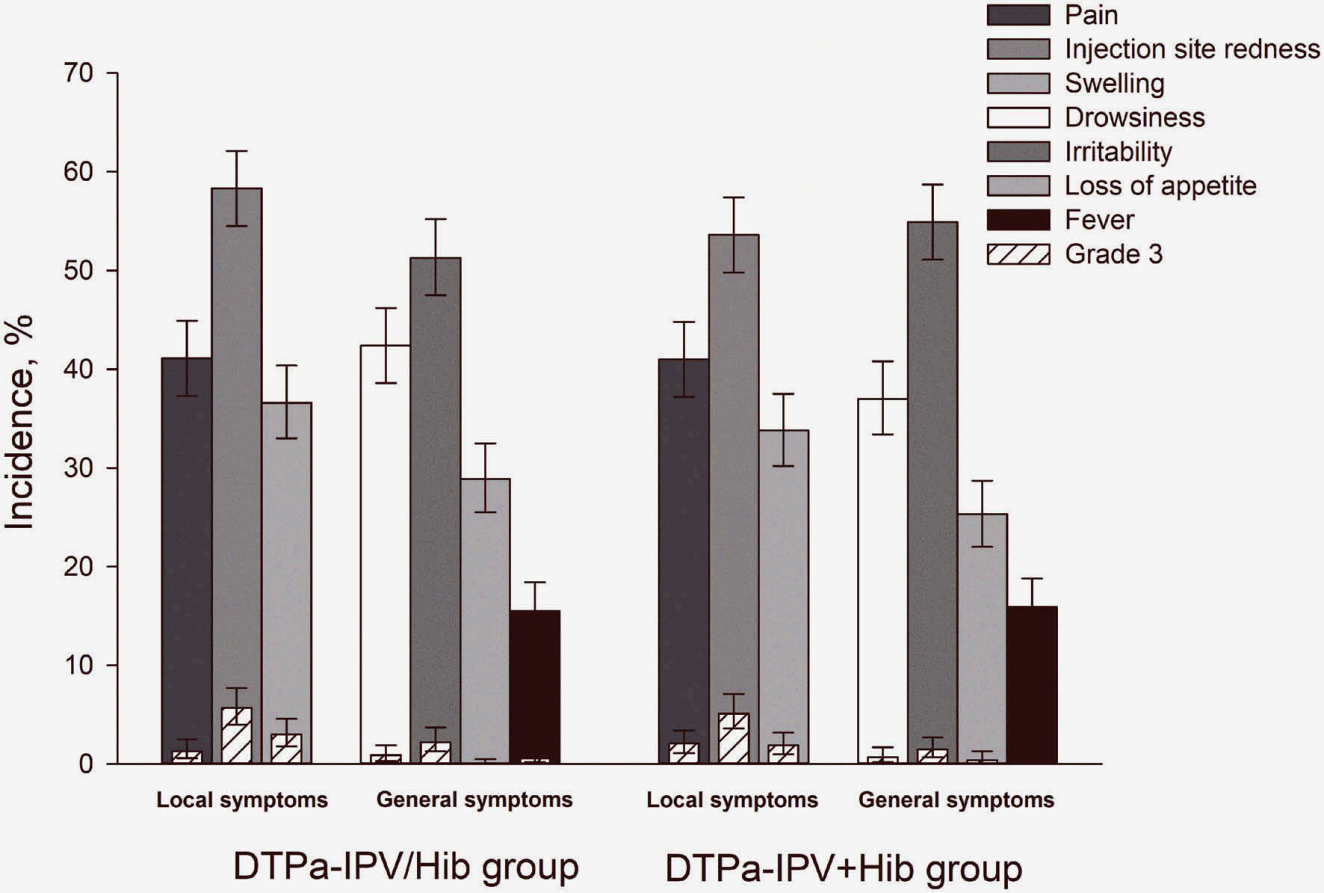
Incidence of solicited local and general symptoms reported up to 4 days post-vaccination (total vaccinated cohort). Infants in DTPa-IPV/Hib group received 3 doses of combined diphtheria-tetanus-acellular pertussis-inactivated poliomyelitis and *Haemophilus influenzae* type b vaccine at 2, 4, 6 months of age and infants in DTPa-IPV+ Hib group received 3 concomitant doses of diphtheria-tetanus-acellular pertussis-inactivated poliomyelitis vaccine and *Haemophilus influenzae* type b vaccine at 2, 4, 6 months of age. The results are reported overall/dose. The error bars indicate 95% confidence intervals. Grade 3 were defined as adverse events preventing normal activity, pain upon limb movement or a spontaneously painful limb, redness and swelling >20 mm in diameter, a tympanic temperature >39.0°C, loss of appetite resulting in not eating at all, drowsiness that prevented normal activity, and irritability/fussiness resulting in crying that cannot be comforted.

The incidence of solicited local and general symptoms was similar between groups (Figure 2). The most common solicited local symptom in both groups was injection site redness, reported after 58.3% and 53.6% of doses in the DTPa-IPV/Hib and DTPa-IPV+Hib groups, respectively; the most common solicited general symptom was irritability, reported after 51.3% and 54.9% of doses in these groups, respectively. Injection site redness and irritability were also the most common local and general grade 3 symptoms, and were reported in 5.7% and 5.1% of doses for redness, and in 2.2% and 1.5% of doses for irritability in the DTPa-IPV/Hib and the DTPa-IPV+Hib groups, respectively (Figure 2). Irritability was also the most common general symptom considered by the investigator to be related to vaccination, and was reported after 32.3% and 34.7% of doses in the DTPa-IPV/Hib and the DTPa-IPV+Hib groups, respectively. Grade 3 irritability considered by the investigator as related to vaccination was reported after 1.5% and 1.0% of doses respectively.

During the 31-day post-vaccination period, at least one unsolicited adverse event (AE) was reported for 58.0% of infants in the DTPa-IPV/Hib group and 54.2% of infants in the DTPa-IPV+Hib group. The most common unsolicited AEs were nasopharyngitis, reported for 16.5% of infants in the DTPa-IPV/Hib group and 12.8% of infants in the DTPa-IPV+Hib group, and upper respiratory tract infections, reported for 15.2% and 12.8% of infants, respectively. Grade 3 unsolicited AEs were reported in 1.8% of infants in the DTPa-IPV/Hib group and in 2.2% of infants in DTPa-IPV+Hib group, and unsolicited AEs possibly related to vaccination were reported in 6.7% and 3.5% of infants, respectively.

Fifty-seven serious AEs (SAEs) were reported in 46 infants (25 and 21 infants in the DTPa-IPV/Hib and DTPa-IPV+Hib groups, respectively); none were considered to be related to vaccination. No fatal SAEs were reported.

## Discussion

In this study, a three-dose primary vaccination with the combined DTPa-IPV/Hib vaccine administered at 2, 4 and 6 months of age induced robust immune responses to all vaccine antigens and had a clinically acceptable safety profile in healthy Korean infants.

The non-inferiority of the DTPa-IPV/Hib vaccine over the DTPa-IPV and Hib vaccines administered concomitantly was demonstrated in terms of the immune response to all vaccine antigens.

Nearly all study participants reached seroprotective antibody levels for anti-D, anti-T, anti-Polio 1, 2 and 3 and anti-PRP. Anti-T and anti-PRP GMCs appeared numerically lower in infants who received the combined DTPa-IPV/Hib vaccine, however seroprotection rates for both antigens were reached by all study participants in both groups. Since all infants in the current study achieved protective anti-T and anti-PRP levels after the third dose, it is unlikely that the lower levels of anti-T and anti-PRP GMCs in the DTPa-IPV/Hib group will have clinical relevance. Our results are in line with previous observations suggesting that lower antibody responses to Hib are induced by combined vaccines containing acellular pertussis compared to separate injections.^6,7,16–18^ Previous studies have shown that lower anti-PRP antibody levels were induced in infants who received 3 primary doses of the combined vaccine as compared to separate injections of DTPa-IPV and Hib, or separate injections of DTPa, oral polio vaccine and Hib.^6,7,18^ In another study conducted in infants primed with a single dose of DTPa vaccine, lower levels of anti-PRP, anti-T, and anti-poliovirus type 1 antibodies were also observed in the infants who received 2 subsequent doses of the combined DTPa-IPV/Hib vaccine compared with separate injections.^16^ Nevertheless, it was also previously suggested that the lower immune responses to the combined vaccine were not associated with an impaired function of the induced antibodies, nor with induction of immune memory against Hib.^17^ The lower levels of anti-T antibodies induced by the combined vaccine in our study are in line with the results of a previous study with a DTPa vaccine combined with Hib compared with DTPa and Hib vaccines given at the same visit but at separate injection sites.^19^

Immune responses to vaccines can be influenced by a number of parameters, including race, ethnicity or other demographic variables.^20–25^ Population-based differences in the response to Hib vaccination have been previously reported. A Hib conjugate vaccine (PRP-conjugated to diphtheria toxoid [DT]) showed high efficacy in Finnish children, but was ineffective in preventing Hib carriage and disease in Alaskan Native infants.^11,14,26,27^ Furthermore, antibody responses to a Hib-tetanus toxoid (TT) conjugate vaccine (Hib-TT) appeared higher among Native South American Indian infants compared to infants in the United States, Europe, or Israel.^10,12,13^ In addition, a previous meta-analysis of Korean studies with Hib conjugate vaccines reported high immunogenicity of Hib-TT conjugate vaccines in Korean infants; after a primary vaccination course with Hib-TT conjugate vaccine given at 2, 4, and 6 months of age, 95.9% (95% CI: 93.5–97.5%) of the infants had anti-PRP antibody levels ≥ 1.0 mg/mL after the third primary dose. ^28^ In a cohort study in Asian infants, all infants achieved anti-PRP antibody concentration of 0.15 mg/mL, following routine vaccination with Hib-TT and DTPa.^29^ In another study, in which Korean infants were vaccinated with DTPa-IPV and Hib, separately or combined, the seroprotection rate for of PRP was 100% for infants achieving seroprotection.^30^ Similarly, in our study, all infants achieved the protective anti-PRP levels following 3 doses of either combined DTPa-IPV/Hib or separate DTPa-IPV and Hib administration. In contrast, in a previous study with a hexavalent DTPa–HBV–IPV/Hib vaccine in a European population, the seroprotection rate for anti-PRP antibodies was 96.0% (95% CI: 93.8–97.5).^31^ These results suggest that the Korean infants may have relatively higher immune response to Hib vaccination compared with Caucasian populations, regardless of the type of vaccine administered. Population-based differences in the response to tetanus, diphtheria, and pertussis antigens have also been suggested.^32^ Indeed, immune responses to all antigens contained in the combined DTPa-Hib vaccine were significantly lower in Belgian vs. Turkish infants (p ≤ 0.001 for all antigens).^33^ Furthermore, in the previous cohort study, Asian infants had higher post-vaccination anti-D and anti-T antibody levels compared to Caucasian infants (5.57 IU/mL vs 3.87 IU/mL and 1.94 IU/mL vs 0.70 IU/mL).^29^ Altogether, these studies and our results suggest that Asian infants, including Koreans, may have enhanced immune responses to DTPa-IPV/Hib vaccine compared to Caucasian populations.

In this study, the incidence of solicited local and general AEs was similar in DTPa-IPV/Hib recipients and in infants who received separate injections of DTPa-IPV and Hib, and grade 3 symptoms were infrequent in both groups, which is in agreement with previous studies.^6,7^ Injection site redness and swelling were the most commonly reported local symptoms and irritability the most common general symptom, which are consistent with the results of previous studies assessing the reactogenicity of DTPa-IPV/Hib,^9,34^ but differs from a study conducted in China, where pain and fever were the most common solicited symptoms after primary and booster vaccinations.^35^ Consistent with previous reports, no SAEs that occurred during the study were considered to be related to the study vaccines by the study investigators.^6,9,35^

Strengths of the trial include high completion rate where nearly all vaccinated children completed the study. A potential limitation of the study was its open design, which is unlikely to have influenced the immunogenicity assessments, but may have biased the safety assessment.

## Conclusions

The results of this study showed that the pentavalent DTPa-IPV/Hib vaccine immunogenicity was non-inferior compared to that of DTPa-IPV and Hib vaccines administered separately. DTPa-IPV/Hib induced protective levels of antibodies against D, T, poliovirus types 1, 2 and 3, and PRP antigens, and seropositive levels against the pertussis antigens. The DTPa-IPV/Hib vaccine had a clinically acceptable safety profile in healthy Korean infants.

## Methods

### Study design

This was a phase III, open-label, randomized study, conducted in 11 centers in Korea between March 2011 and February 2012.

Participants were randomized (1:1) to receive either the combined DTPa-IPV/Hib vaccine (DTPa-IPV/Hib group) or separate DTPa-IPV and Hib vaccines (DTPa-IPV+Hib group) at 2, 4 and 6 months of age. According to the recommendations of The Korean Society of Pediatric Infectious Disease on immunization schedule, all infants received the pneumococcal nontypeable *H. influenzae* protein D conjugate vaccine (PHiD-CV; *Synflorix*, GSK) and the rotavirus vaccine (*Rotarix*, GSK) in a staggered manner. PHiD-CV was administered at 6 weeks, 3.5 and 5.5 months of age, while the rotavirus vaccine was administered at 6 weeks and 3.5 months of age.

The randomization list was generated using a program developed for use in the Statistical Analysis Software (SAS®, SAS Institute Inc., Cary, NC, United States) by GSK. The treatment allocation at the site was done with GSK´s central randomization system on internet (SBIR). As the number of injections was different between groups, the study was conducted in an open manner.

Written informed consent was obtained from the parent or the legally acceptable representative of each infant. The study was conducted according to the Declaration of Helsinki, Good Clinical Practice and Korean laws and regulations.

The study protocol, the informed consent, and all documents requiring pre-approval were reviewed and approved by the Institutional Review Board of the Catholic Medical Center of the Catholic University of Korea (approval number XC10BDMT0127V).

The summary of results for this study (GSK study number 114260 – NCT01309646) is available on the GSK Clinical Study Register and can be accessed at www.gsk-clinicalstudyregister.com.

### Study objectives

The primary objective of the study was to demonstrate non-inferiority of the combined DTPa-IPV/Hib vaccine compared to the concomitant administration of DTPa-IPV and Hib vaccines in terms of immune response to all vaccine antigens 1 month after the third primary dose.

The secondary objectives included the assessment of the immune response induced by DTPa-IPV/Hib versus DTPa-IPV and Hib in terms of seroprotection/seropositivity rates and antibody GMCs/GMTs to all vaccine antigens and in terms of vaccine response to pertussis antigens 1 month after the third primary dose; the assessment of the safety and reactogenicity of the study vaccines in terms of occurrence of solicited symptoms, unsolicited AEs, and SAEs.

### Study participants

Participants were healthy infants aged 42–69 days at the time of first vaccination, born after a gestation period of 37–42 weeks.

Infants in care or infants who received any investigational/non-registered drug or vaccine other than the study vaccines (with exception of hepatitis B and Bacillus Calmette-Guérin vaccines) within 30 days preceding the first dose, or planned use during the study period (hepatitis B and influenza vaccines were allowed at least 7 days before or 30 days after the administration of the DTPa vaccine), or those who received immunoglobulins, blood products and/or immunosuppressants were excluded. Infants with evidence of diphtheria, tetanus, pertussis, poliomyelitis and Hib vaccination or disease, family history of immunodeficiency, immunosuppressive or immunodeficient condition, major congenital defects, serious chronic illness, history of neurological disorders, seizures, reaction or hypersensitivity likely to be exacerbated by any component of the vaccines, acute disease, or fever at the time of enrollment were also ineligible.

### Study vaccines and administration

Each 0.5 mL dose of DTPa-IPV/Hib contained ≥ 30 IU DT, ≥ 40 IU TT, 25 μg PT, 25 μg FHA, 8 μg PRN, 40 D antigen units (DAgU) poliovirus type 1, 8 DAgU poliovirus type 2, 32 DAgU poliovirus type 3, 10 μg purified Hib capsular polysaccharide (PRP), conjugated to TT (20–40 μg), lactose, and 0.5 mg aluminum as salts. The DTPa-IPV and Hib vaccines contained the same antigen constituents as the DTPa-IPV/Hib vaccine.

The composition of pneumococcal and rotavirus vaccines has been previously described.^36,37^

DTPa-IPV/Hib, DTPa-IPV and Hib were administered intramuscularly in the anterolateral side of the thigh (right thigh for DTPa-IPV/Hib and DTPa-IPV, and left thigh for Hib).

### Immunogenicity assessment

Blood samples were collected pre-vaccination and one month post-dose 3.

Anti-D and anti-T antibodies were evaluated by an enzyme-linked immunosorbent assay (ELISA), with seroprotection defined as antibody concentrations ≥ 0.1 IU/mL. Anti-PT, anti-FHA, and anti-PRN antibodies were measured by ELISA, with seropositivity defined as antibody concentrations above the cut-off of 5 ELISA Units per mL (EL.U/mL). Antibodies against poliovirus types 1, 2 and 3 were determined by a virus micro-neutralization test,^38^ with seroprotection defined as antibody titers ≥ 8 median effective dose (ED_50_). Anti-PRP antibodies were measured by ELISA, with antibody concentrations ≥ 0.15 µg/mL considered protective and ≥ 1 µg/mL considered indicative of long-term protection.

### Safety assessment

Solicited local (pain, injection site redness, swelling) and general symptoms (fever [tympanic temperature ≥ 37.5°C], drowsiness, irritability/fussiness, loss of appetite) were recorded up to 4 days after each dose of DTPa-IPV/Hib or DTPa-IPV and Hib vaccines. Unsolicited AEs were recorded up to 31 days after vaccination. SAEs were recorded throughout the study.

Grade 3 symptoms were defined as AEs preventing normal activity, pain upon limb movement or a spontaneously painful limb, injection site redness and swelling >20 mm in diameter, a tympanic temperature >39.0°C, loss of appetite resulting in not eating at all, drowsiness that prevented normal activity, and irritability/fussiness resulting in crying that cannot be comforted.

All solicited local reactions at injection site were considered causally related to vaccination. Causality of all other symptoms and AEs/SAEs was assessed by the investigator.

### Statistical analysis

Assuming a drop-out rate of 20%, a sample size of 450 participants (225 participants per group) was calculated to meet all endpoints with an overall power of 90%.

The primary safety analysis was based on the TVC, which included all participants who received at least one vaccine dose. Immunogenicity was assessed on the ATP cohort for immunogenicity, which included all participants who met eligibility criteria, received at least one dose of DTPa-IPV/Hib or DTPa-IPV and Hib, with known ATP administration site, had not received a vaccine not specified or forbidden in the protocol, complied with the procedures and intervals defined in the protocol, did not meet any elimination criteria, did not receive any product or have any medical condition leading to exclusion, and had available data for the immunogenicity endpoints.

Non-inferiority of the immune response induced by DTPa-IPV/Hib versus DTPa-IPV and Hib was demonstrated if the ULs of the standardized asymptotic 95% CIs on the group differences (DTPa-IPV+Hib minus DTPa-IPV/Hib group) in percentages of infants seroprotected against diphtheria, tetanus, poliovirus types 1, 2 and 3, and Hib were all ≤ 10%, and if the ULs of the 95% CIs on the group ratios (DTPa-IPV+Hib over DTPa-IPV/Hib group) of anti-PT, anti-FHA and anti-PRN GMCs were all ≤ 1.5.

The GMC/GMT calculations were performed by taking the anti-log of the mean of the log_10_ titer/concentration transformations, and were calculated with their exact 95% CIs for each antigen. Seropositivity rates against PT, FHA and PRN, and seroprotection rates against D, T, PRP and poliovirus types 1, 2 and 3 were calculated with 95% CIs. Percentage of infants with anti-D and anti-T antibody concentrations ≥ 1.0 IU/mL, and anti-PRP antibody concentrations ≥ 1.0 μg/mL were calculated with 95% CIs. Vaccine response rates to PT, FHA and PRN were calculated with exact 95% CI, 1 month after the third vaccine dose.

The statistical analyses were performed using SAS® version 9.2 and StatXact-8.1 procedure for SAS.

## Trademark

*Infanrix, Hiberix, Synflorix, and Rotarix* are trademarks of the GSK group of companies.

## Abbreviations

AE: adverse event
anti-D: anti-diphtheria
anti-T: anti-tetanus
ATP: according-to-protocol
CI: confidence interval
DAgU: D antigen units
DT: diphtheria toxoid
DTPa: combined vaccine against diphtheria, tetanus, pertussis
DTPa-IPV: combined vaccine against diphtheria, tetanus, pertussis and poliomyelitis
DTPa-IPV/Hib: combined pentavalent vaccine against diphtheria, tetanus, pertussis, poliomyelitis and Hib
ED50: median effective dose
ELISA: enzyme-linked immunosorbent assay
EL.U/mL: ELISA units per milliliter
FHA: filamentous hemagglutinin
GMC: geometric mean concentration
GMT: geometric mean titer
Hib: *Haemophilus influenzae* type b
IU: international units
PRN: pertactin
PRP: polyribosyl-ribitol phosphate
PT: pertussis toxoid
SAE: serious adverse event
TT: tetanus toxoid
TVC: total vaccinated cohort
UL: upper limit.
